# Sustainability of pneumococcal conjugate vaccination in Ghana: a cost-effectiveness analysis in the context of donor transition

**DOI:** 10.3389/fpubh.2024.1383668

**Published:** 2024-08-01

**Authors:** Abdul-Mumin Ibrahim, Richmond Owusu, Justice Nonvignon

**Affiliations:** Department of Health Policy, Planning and Management, School of Public Health, College of Health Sciences, University of Ghana, Accra, Ghana

**Keywords:** cost-effectiveness, streptococcus pneumonia, vaccination, Gavi transition, Ghana

## Abstract

**Background:**

Streptococcus pneumonia is responsible for 18% of infant deaths in Ghana. With co-financing from Gavi in 2012, Ghana introduced the PCV13 into the childhood immunization programme to reduce the burden of Streptococcus pneumonia. However, Ghana will graduate to the Gavi fully self-financing phase in 2026, when the nation assumes full responsibility of paying for the PCV13. This research aims to evaluate the health impact and cost-effectiveness of PCV13 immunization in Ghana since its implementation and after the cessation of support from Gavi.

**Methods:**

We used the UNIVAC tool to evaluate two main scenarios of cost-effectiveness, from vaccine introduction (2012–2025) and after Gavi transition (2026–2031) in comparison with no vaccination. The sources of data include national data, international estimates and expert opinion. Cost was considered from both the government and societal perspectives. We discounted health outcomes at 3%. Currency values were stated in US Dollars. We tested the robustness of the base case results by performing scenario and sensitivity analyses.

**Results:**

PCV13 will reduce the pneumococcal disease burden by 48% from 2012 to 2031. The vaccination programme costs are USD 130 million and USD 275 million in 2012–2025 and 2026–2031 respectively. It also has a budget impact of USD 280 million for the 2026–2031 period from the perspective of government. The incremental cost-effectiveness ratios are USD 89 and USD 73 respectively from the perspectives of government and society in 2012–2025. The incremental cost-effectiveness ratios are USD 530 and USD 510 respectively from the perspectives of government and society in 2026–2031.

**Conclusion:**

The PCV13 vaccination programme in Ghana is cost-effective at 50% GDP per capita threshold even when Gavi withdraws co-financing support from 2026 onwards.

## 1 Introduction

Pneumococcal disease is the leading global cause of mortality from contagious diseases in children under 5 years (1). An estimated 920,000 children under than 5 years died from pneumonia in 2015 (2). Africa and Asia are reported to have the highest burden of invasive pneumococcal disease (IPD) (3). Pneumonia is responsible for 18% of deaths in children under 5 years in Ghana (4). Annual pneumonia and meningitis cases are estimated to be 6,441 and 286 respectively in Northern Ghana alone. The pneumococcal disease has a substantial economic burden on families in Ghana estimated at USD 777 per case (5).

The pneumococcal conjugate vaccine (PCV) gives immunity to children from Streptococcus pneumonia and its associated conditions. The World Health Organization (WHO) therefore recommended PCV to be included in childhood immunization programmes since 2007. Consequently, PCV immunization has been implemented in more than 140 countries globally (1, 2, 6). The implementation of PCV immunization has led to a significant decline in the global burden of Streptococcus pneumoniae. In Africa, pneumococcal deaths have fallen by 63% from 447,000 to 166,000 between 2000 and 2015 (7, 8).

Gavi, the Vaccine Alliance has supported many African countries to introduce PCV where countries initially pay USD 0.20 per dose whereas Gavi pays the remaining amount (8). As the GDP per capita of countries increase, they move from self-financing phase to preparatory transition, accelerated transition and finally to the fully self-financing phase. Countries pay a higher proportion of the full price upon graduating to each stage. They eventually pay the full price for the PCV when they reach the fully self-financing stage (8).

PCV7 was the first pneumococcal conjugate vaccine that was introduced in 2000 but was never introduced in Ghana. Subsequently, PCV10 were introduced in 2009 (3). To reduce the burden of the pneumococcal disease in children under 5 years, Ghana introduced the PCV13 into the infant immunization programme in 2012 with co-financing support from Gavi (5). The current PCV13 coverage in Ghana is estimated at 97% following a dosing schedule of 6, 10, and 14 weeks, respectively (9). However, the country is currently faced with substantial reduction in official development assistance (ODA) as a result of attaining a lower middle-income country (LMIC) status since 2010. Consequently, financial support from development partners fell by 30% between 2011 and 2013 (10). The cost of PCV13 may have significant budget impact for Government of Ghana, and the ability of the government to afford this have consequences for the coverage levels of this vaccine for children.

The withdrawal of development partner support adversely affects a country's health system in terms of service delivery, health technologies and finance. This becomes more problematic in situations where financial and technical assistance are simultaneously withdrawn as Ghana is currently experiencing (11). A cross-programmatic efficiency analysis undertaken by the WHO in 2017 revealed that Ghana had defaulted on two occasions regarding co-financing commitments to Gavi. Ghana also moved to the Gavi accelerated transition phase in 2021 which will span a period of 5 years before getting to fully self-financing phase (9). This implies that Ghana might fully finance the childhood vaccines programme from 2026 onwards at a time the country is facing economic challenges coupled with limited fiscal space for health financing (9).

While literature is replete on the incidence of Streptococcus pneumoniae in Ghana and the African meningitis belt in general (4, 5, 12), little is known about the cost-effectiveness of PCV vaccination in Ghana. There is therefore the need to generate empirical evidence on the cost-effectiveness of PCV vaccination to provide decision-makers with the needed evidence to prioritize the intervention, considering the health and economic impact of this investment in an era of donor transition in Ghana. We therefore aimed to evaluate the health effects and cost-effectiveness of PCV13 in Ghana since introduction and beyond the Gavi transition.

## 2 Methods and materials

### 2.1 Study population and setting

This is a national-level study conducted in Ghana starting from the implementation of PCV13 vaccination programme in 2012 to 2025 and beyond the GAVI transition (2026 to 2031). Ghana is located on the coast of West Africa between latitude 5°33′ North and 0°12′ West and longitude 5.550° North and 0.200° West with a landmass of 238,537 square kilometers. Ghana is bordered in the North and Northwest by Burkina Faso, in the East by Togo, in the West by Cote d'Ivoire and in the South by the Gulf of Guinea.

The 2021 Population and Housing Census records the total population of Ghana to be 30,832,019. Ghana has sixteen administrative regions which include: Upper East, North East, Upper West, Savannah, Northern, Bono, Bono East, Ahafo, Ashanti, Oti, Volta, Eastern, Western, Western North, Central, and Greater Accra Regions (13). The Census also records the population of children below the age of 5 years in Ghana as 3,773,723. This study simulates health outcomes and cost of PCV immunization in children under 5 years for 20 successive birth cohorts.

### 2.2 Model and perspective of analysis

We used the UNIVAC (version 1.4) model to evaluate the cost-effectiveness of PCV13 compared to a scenario of no vaccination programme in Ghana from 2012 to 2025 and from 2026 to 2031. UNIVAC is a proportionate outcomes model which was developed in the London School of Hygiene and Tropical Medicine (14). It is a Microsoft Excel spreadsheet software designed to calculate the incremental cost-effectiveness ratios (ICERs) and other pertinent indicators for Haemophilus Influenza B (HiB), Rotavirus vaccine (RVV), and Pneumococcal Conjugate Vaccine (PCV).

The input parameters for the UNIVAC model include demography, burden of disease, health services utilization and treatment costs, vaccination coverage, vaccine efficacy and vaccination costs. The cost per DALY averted denotes the weighted combination of morbidity and mortality effects of the PCV immunization programme and hence, it is the primary outcome measure of this study. Disability-adjusted life years (DALYs) are more preferable outcome measures in LMICs and are therefore utilized in this study. The structure and details of the UNIVAC model have been published elsewhere (14, 15).

The manufacturers of PCV10 and PCV13 recommend three primary doses at an interval of at least 4 weeks, plus a booster dose at least 6 months after the third dose. The first dose can be given as early as 6 weeks of age whilst the booster dose is given preferably between 9 and 15 months of age. Alternatively, two primary doses can be administered 2 months separately, starting at 2 months of age. This is followed by a booster dose at least 6 months after the second dose for PCV10 and at 11 to 15 months of age for PCV13 (1).

We compared two periods of PCV immunization of children below 5 years in Ghana. In the first period, we examined the health effects and costs of the current PCV immunization programme from introduction (2012 to 2025) with a hypothetical scenario of “No PCV immunization.” In the second period, we compared the health effects and costs of the PCV immunization beyond the Gavi transition (2026 to 2031) with a scenario of “No immunization” within the same period of time. In the first scenario, cost-effectiveness is analyzed based on only the price per dose Ghana pays during the 14 years of Gavi co-financing support. The second scenario assesses cost-effectiveness according to the full cost Ghana may incur to continue with PCV immunization 6 years without Gavi co-financing support, which coincides with the deadline of Universal Health Coverage (UHC) Roadmap in Ghana. The analysis considers cost from the perspectives of both government and society. The second scenario gives a clearer picture of cost-effectiveness as the price per dose will dramatically increase when support from Gavi discontinues.

### 2.3 Analytic framework of cost-effectiveness of PCV vaccination

**Figure 1:**
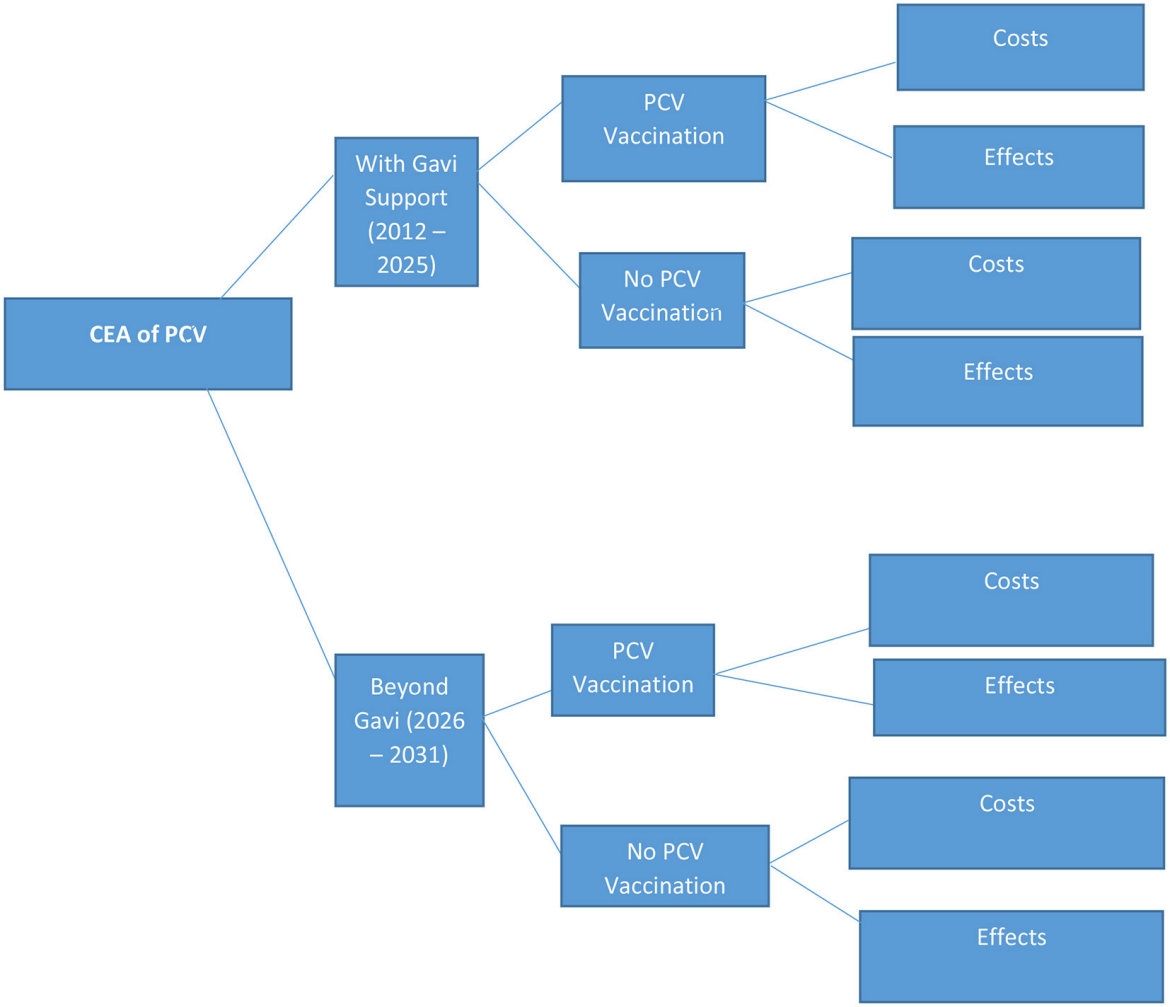
Analytic framework of cost-effectiveness of PCV vaccination.

Figure 1 illustrates the analytic framework for this study. The framework compares two periods of PCV immunization of children below 5 years in Ghana. The first period examines the current PCV immunization programme from introduction (2012 to 2025) with a hypothetical scenario of “No PCV immunization.” The second period compares PCV immunization beyond the Gavi transition (2026 to 2031) with a scenario of “No immunization” in Ghana within the same period of time.

The health effects and costs of vaccination are estimated under two scenarios. In the first scenario, cost-effectiveness is analyzed based on only the price per dose Ghana pays during the 14 years of Gavi co-financing support. The second scenario assesses cost-effectiveness according to the full cost Ghana may incur to continue with PCV immunization 6 years without Gavi co-financing support. The analysis considers cost from the perspectives of both government and the society. The second scenario really gives a clearer picture of cost-effectiveness as the price per dose will dramatically increase when support from Gavi discontinues.

### 2.4 Disease burden and healthcare utilization

We considered the various disease conditions of Streptococcus pneumonia such as acute otitis media (AOM), pneumonia (non-severe), pneumonia (severe), meningitis, meningitis (sequelae), non-severe non-pneumonia non-meningitis (NPNM) and severe NPNM. For each category, there were estimates for the number of cases, outpatient visits, inpatient admissions and deaths. Notably, this study did not attribute deaths to pneumonia (non-severe) and non-severe NPNM (16–18).

We also examined outcomes for outpatient pneumonia (non-severe), inpatient pneumonia (severe), and Streptococcus meningitis. Pneumonia (non-severe) was assumed to result in outpatient visit followed by recovery. Severe pneumonia on the other hand results in inpatient admission followed by recovery or death. Streptococcus meningitis results in inpatient admission followed by recovery with or without sequelae or death (16–18).

Data was obtained from the 2014 Demographic and Health Survey (DHS) and the 2017 Multiple Indicator Cluster Survey (MICS) (19). Data from international estimates, particularly from the Maternal and Child Epidemiology Estimation (MCEE) by Wahl et al. (3), were used in instances where there was paucity of local data on some disease episodes at the national level. To calculate years of life lost (YLLs), data on number of deaths and life expectancy were obtained from the annual data of the United Nations World Population Prospects (UNWPP).

### 2.5 Estimates for disability-adjusted life years

Disability weights on Streptococcus pneumonia were obtained from the Global Burden of Disease (GBD) study conducted by Salomon et al. (20). The GBD disability weights allows for the use of a common data source to evaluate health states (20).

### 2.6 Vaccination coverage

In Ghana, PCV13 is administered along with the first and third doses of diphtheria/tetanus/pertussis (DTP) vaccine during 2, 3 and 4 months of age. We accessed coverage levels of PCV13 from the immunization coverage database of the WHO and UNICEF with the assumption that it was constant during the course of the 20 birth cohorts (21).

### 2.7 Vaccine efficacy

Data on vaccine efficacy was obtained from Lucero et al. (22). The evidence from this systematic review is reported to be of high quality based on Cochrane risk of bias tool. The vaccine efficacy after the first dose is 29%. However, the vaccine efficacy after the second and booster doses is 58%. In the UNIVAC model, the period of efficacy is assumed to be lifelong.

### 2.8 Vaccination programme costs

The data on price per dose and costs of delivery were taken from the database of the UNICEF Vaccine Supply Division (23). For the purpose of this study, we estimated the total vaccination cost as a sum of vaccine price per dose, fixed price assumption for safety box (USD 0.03), estimated wastage (5%) and incremental health system costs per dose. As the vaccination programme is already functioning under the Expanded Programme on Immunization (EPI), cold chain and other start-up costs were excluded as they are sunk costs. Data on cost of treatment was obtained from a study conducted by Kobayashi et al. (5).

### 2.9 Outpatient visits and inpatient admissions costs

We only applied costs to cases that sought care at the health facility. We also applied a discount rate of 3% to future health outcomes based on WHO recommendation (24). We reported all the monetary units in US Dollars with 2022 Ghana Cedis equivalent.

### 2.10 Data quality control

Any error identified was noted and validated by comparing with other datasets in order to get the right measure of the parameter. This enhanced the reliability in the data. It also produced a credible analysis of the data. The data extracted is stored into hard disk drive and protected with password to prevent any tampering which might affect consistency in the results. A copy of the data is also saved on Google Drive to enable access to the data to forestall any unforeseen circumstance that might lead to loss of data. The inputs can be retrieved through such methods as systematic reviews, meta-analysis and published literature, among others.

Table 1 shows the inputs for the base case analysis.

**Table 1:** Inputs for base case parameters.

**Input parameter**	**Mid value**	**Low**	**High**	**Source**
**Acute otitis media**				
Incidence rate < 5 years (per 100,000)	11,555	11,422	11,687	(18)
Outpatient visits < 5 years (per 100,000)	5,801	5,734	5,867	(19)
Hospitalizations < 5 years (per 100,000)	277	127	351	(16)
Deaths < 5 years (per 100,000)	91	85	98	(18)
**Streptococcus pneumonia (non-severe)**				
Incidence rate < 5 years (per 100,000)	851	796	1,038	(3)
Outpatient visits < 5 years (per 100,000)	427	400	521	(19)
**Streptococcus pneumonia (severe)**				
Incidence rate < 5 years (per 100,000)	546	409	623	(3)
Outpatient visits < 5 years (per 100,000)	274	206	313	(19)
Hospitalizations < 5 years (per 100,000)	274	206	313	(19)
Deaths < 5 years (per 100,000)	64	46	67	(3)
**Streptococcus meningitis**				
Incidence rate < 5 years (per 100,000)	16	6	34	(3)
Outpatient visits < 5 years (per 100,000)	8	3	17	(19)
Hospitalizations < 5 years (per 100,000)	8	3	17	(19)
Deaths < 5 years (per 100,000)	9	3	19	(3)
Sequelae cases < 5 years (per 100,000)	3	1	7	(17)
**Streptococcus NPNM (non-severe)**				
Incidence rate < 5 years (per 100,000)	48	18	104	(3)
Outpatient visits < 5 years (per 100,000)	24	9	52	(19)
**Streptococcus NPNM (severe)**				
Incidence rate < 5 years (per 100,000)	18	7	39	(3)
Outpatient visits < 5 years (per 100,000)	9	3	20	(19)
Hospitalizations < 5 years (per 100,000)	9	3	20	(19)
Deaths < 5 years (per 100,000)	8	3	17	(3)
**Disability weights**				
Acute otitis media	1%	1%	2%	(20)
Streptococcus pneumonia (non-severe)	5.1%	3.2%	7.4%	(20)
Streptococcus pneumonia (severe)	13.3%	8.8%	19%	(20)
Streptococcus meningitis	13.3%	8.8%	19%	(20)
Streptococcus NPNM (non-severe)	5.1%	3.2%	7.4%	Assumption (same as non-severe pneumonia)
Streptococcus NPNM (severe form)	13.3%	8.8%	19%	Assumption (same as meningitis)
Streptococcus meningitis sequelae	26%	15.3%	36.4%	Assumption
**Vaccine coverage**				
Dose 1 (with DTP1)	94%	85%	100%	(21)
Dose 2 (with DTP2)	94%	84%	100%	(21)
Dose 3 (with DTP3)	93%	84%	100%	(21)
**Vaccine efficacy**				
1st Dose	29%	14.5%	37.5%	Assumption (half of full efficacy)
**Acute otitis media**				
2nd Dose	58%	29%	75%	(22)
Booster Dose	58%	29%	75%	(22)
**Mean Duration of Illness (in days)**				
Acute Otitis Media	7	6	9	Expert opinion
Streptococcus pneumonia (non-severe)	7	6	9	Expert opinion
Streptococcus pneumonia (severe)	10	7	21	Expert opinion
Streptococcus meningitis	10	7	21	Expert opinion
Streptococcus NPNM (non-severe)	7	6	9	Expert opinion
Streptococcus NPNM (severe form)	10	7	21	Expert opinion
**Vaccine programme and health system costs**				
Vaccine price per dose (2012–2022)	USD 2.90			(23)
Vaccine price per dose (2023–2025)	USD 2.75			(23)
Vaccine price per dose (After 2025)	USD 14.50			(23)
Syringe price per dose	USD 0.03	USD 0.03	USD 0.03	(23)
Vaccine and syringe wastage	5%	5%	5%	Assumption
**Government Cost per Outpatient**				
Acute Otitis Media	USD 1.3	USD 0.00	USD 2.9	(5)
Streptococcus pneumonia (non-severe)	USD 1.3	USD 0.00	USD 2.9	(5)
Streptococcus pneumonia (severe)	USD 1.3	USD 0.00	USD 2.9	(5)
Streptococcus meningitis	USD 1.3	USD 0.00	USD 2.9	(5)
Streptococcus NPNM (non-severe)	USD 1.3	USD 0.00	USD 2.9	(5)
Streptococcus NPNM (severe)	USD 1.3	USD 0.00	USD 2.9	(5)
Streptococcus meningitis sequelae	USD 1.3	USD 0.00	USD 2.9	(5)
**Government cost per hospitalization**				
Acute otitis media	USD 128.5	USD 115	USD 200	(5)
Streptococcus pneumonia (severe)	USD 128.5	USD 115	USD 200	(5)
Streptococcus meningitis	USD 141.1	USD 112.6	USD 324	(5)
Streptococcus NPNM (severe form)	USD 128.5	USD 115	USD 200	(5)
**Household cost per outpatient**				
Acute otitis media	USD 12.7	USD 3.2	USD 47.5	(5)
Streptococcus pneumonia (non-severe)	USD 12.7	USD 3.2	USD 47.5	(5)
Streptococcus pneumonia (severe)	USD 12.7	USD 3.2	USD 47.5	(5)
Streptococcus meningitis	USD 12.7	USD 3.2	USD 47.5	(5)
Streptococcus NPNM (non-severe)	USD 12.7	USD 3.2	USD 47.5	(5)
Streptococcus NPNM (severe form)	USD 12.7	USD 3.2	USD 47.5	(5)
Streptococcus meningitis sequelae	USD 12.7	USD 3.2	USD 47.5	(5)
**Household cost per hospitalization**				
Streptococcus pneumonia (severe)	USD 141.1	USD 112.6	USD 324	(5)
Streptococcus meningitis	USD 141.1	USD 112.6	USD 324	(5)
Streptococcus NPNM (severe form)	USD 141.1	USD 112.6	USD 324	(5)

## 3 Results

### 3.1 Health impact of PCV13 vaccination in Ghana

PCV13 vaccination is estimated to avert 3,666,153 discounted episodes of total pneumococcal illness including 1,840,409 outpatient visits, 159,890 hospitalizations and 40,317 deaths from 2012 to 2025. While 1,692,582 discounted episodes of total pneumococcal illness including 849,676 outpatient visits, 73,818 hospitalizations and 19,024 deaths would be averted from 2026 to 2031. In particular, PCV13 vaccination would avert 91% of Acute Otitis Media cases which constitutes 89% of cases of Streptococcus pneumonia. Severe pneumonia contributes 37% to the total pneumococcal deaths and hence, PCV13 vaccination will avert nearly 50% of severe pneumonia deaths from 2012 to 2031. In total, the PCV13 vaccination programme will reduce the pneumococcal disease burden by 48% from 2012 to 2031 compared to a scenario of no vaccination.

Table 2 shows the results of the estimated reduction in the burden of streptococcus pneumonia in children under-5 years between the period of 2012–2025 and 2026–2031.

**Table 2:** Estimated reduction in the burden of streptococcus pneumonia in Ghana.

	**2012–2025**			**2026–2031**		
**Disease burden**	**No vaccine**	**With vaccine**	**Averted**	**No vaccine**	**With vaccine**	**Averted**
Total cases	7,659,030	3,992,877	3,666,153	3,536,005	1,843,423	1,692,582
Acute otitis media	6,787,591	3,538,571	3,249,020	3,133,681	1,633,680	1,500,001
Pneumococcal pneumonia (non-severe)	500,251	260,796	239,455	230,955	120,404	110,551
Pneumococcal pneumonia (severe)	321,145	167,422	153,723	148,266	77,295	70,970
Pneumococcal meningitis	9,422	4,912	4,510	4,350	2,268	2,082
Pneumococcal NPNM (non-severe)	28,449	14,831	13,618	13,134	6,847	6,287
Pneumococcal NPNM (severe)	10,701	5,579	5,122	4,940	2,576	2,365
Meningitis sequelae	1,471	767	704	679	354	325
*Total outpatient visits*	3,844,388	2,004,424	1,840,409	1,775,074	925,398	849,676
Acute otitis media	3,407,371	1,776,362	1,631,008	1,573,108	820,107	753,000
Pneumococcal pneumonia (non-severe)	251,126	130,919	120,207	115,939	60,443	55,497
Pneumococcal pneumonia (severe)	161,215	84,046	77,169	74,429	38,802	35,627
Pneumococcal meningitis	4,730	2,466	2,264	2,184	1,138	1,045
Pneumococcal NPNM (non-severe)	14,281	7,445	6,836	6,593	3,437	3,156
Pneumococcal NPNM (severe)	5,372	2,801	2,571	2,480	1,293	1,187
Meningitis sequelae	739	385	354	341	178	163
*Total inpatient admissions*	334,029	174,139	159,890	154,214	80,396	73,818
Acute otitis media	162,713	84,827	77,886	75,121	39,163	35,958
Pneumococcal pneumonia (severe)	161,215	84,046	77,169	74,429	38,802	35,627
Pneumococcal meningitis	4,730	2,466	2,264	2,184	1,138	1,045
Pneumococcal NPNM (severe)	5,372	2,801	2,571	2,480	1,293	1,187
*Total deaths*	84,228	43,910	40,317	39,743	20,719	19,024
Acute otitis media	44,746	23,327	21,814	21,114	11,007	10,106
Pneumococcal pneumonia (severe)	31,356	16,347	15,009	14,796	7,713	7,082
Pneumococcal meningitis	4,299	2,241	2,051	2,028	1,057	971
Pneumococcal NPNM (severe)	3,827	1,995	1,832	1,806	941	864
*DALYs (Discounted)*	2,623,383	1,370,135	1,253,248	1,049,523	548,147	501,376
*DALYs (Undiscounted)*	6,981,303	3,641,831	3,339,472	2,556,397	1,333,614	1,222,783

### 3.2 Total healthcare costs

Compared with a scenario of no vaccination, averted outpatient visits and hospitalizations will lead to a reduction in healthcare cost by USD 18,350,140 and USD 9,504,246 in 2012–2025 and 2026–2031 respectively from the perspective of government. The healthcare costs averted from the societal perspective are USD 37,897,274 and USD 19,628,461 respectively in 2012–2025 and 2026–2031 as shown in Table 3.

**Table 3:** Total healthcare costs.

	**2012–2025**			**2026–2031**		
	**Without**	**With**	**Averted**	**Without**	**With**	**Averted**
*Total Gov't costs*	38,403,054	20,052,915	18,350,140	19,890,424	10,386,178	9,504,246
Outpatient	3,186,270	1,663,774	1,552,496	1,650,292	861,733	788,559
Hospitalizations	35,216,785	13,389,141	16,827,644	18,240,132	9,524,446	8,715,686
*Total societal health costs*	79,311,172	41,413,898	37,897,274	41,078,317	21,449,856	19,628,461
Outpatient	40,767,792	21,287,709	19,480,083	21,115,213	11,025,726	10,089,487
Hospitalizations	38,543,380	20,126,188	18,417,191	19,963,104	10,424,130	9,538,974

### 3.3 Cost of vaccination programme

Table 4 shows the total costs of the PCV vaccination programme to be USD 130 million and USD 275 million respectively for the periods of 2012–2025 and 2026–2031 at an annual discount rate of 3%. The PCV13 vaccination programme will have incremental costs of approximately USD 2 million and USD 2.6 million for the periods of 2012–2025 and 2026–2031 respectively after discounting at 3% per year. Also, the cost of vaccine introduction and DALYs averted are the same from the perspectives of government and society.

**Table 4:** Base cost-effectiveness.

	**2012–2025**		**2026–2031**	
***Summary of base case cost***	**Government perspective**	**Societal perspective**	**Government perspective**	**Societal perspective**
*Net cost of vaccination*	111,467,257	91,920,123	265,649,021	255,524,806
Cost of vaccine introduction	129,817,397	129,817,397	275,153,267	275,153,267
Health service costs averted	18,350,140	37,897,274	9,504,246	19,628,461
**Base case cost-effectiveness results**				
Cost per DALY averted (discounted)	118	97	530	510
Cost per DALY averted (undiscounted)	40	33	234	225

### 3.4 Cost-effectiveness of PCV13 vaccination

Table 4 shows that the discounted costs required to avert one DALY from the perspectives of government and society are USD 118 and USD 97 respectively, representing 5% and 4% of 2022 GDP per capita (USD 2,445.30) from 2012 to 2025. However, the discounted costs for averting one DALY from 2026 to 2031 are USD 530 and USD 510 representing 22% and 21% of GDP per capita respectively from the perspectives of government and society.

The undiscounted ICERs show costs per DALY averted in 2012–2025 to be USD 40 and USD 33 respectively from the perspectives of government and society. In the case of the 2026–2031 period, the undiscounted ICERs are USD 234 and USD 225 from the government and societal perspectives respectively.

From the base case analysis, the PCV13 immunization programme in Ghana is highly cost-effective with respect to the GDP per capita of Ghana (25). This is true for both scenarios with co-financing support from Gavi and even if Gavi exits in 2026 as envisaged.

### 3.5 Scenario analysis

Table 5 shows that the parameter with the highest ICER is low vaccine efficacy, followed by low disease incidence and low treatment cost both from the perspectives of government and society. All other parameters have the same ICER as the base case (most probable) scenario from both government and societal perspectives. Furthermore, ICERs obtained in the period 2012–2025 are lower than those obtained within the period of 2026–2031 from both perspectives. When individual parameters are varied across scenarios for uncertainty, the highest costs per DALY averted are USD 1,079 and USD 1,058 representing 44% and 43% of GDP per capita respectively from the perspectives of government and society.

**Table 5:** Incremental cost-effectiveness ratio for scenario analysis.

**Parameters**	**2012–2025**		**2026–2031**	
**Scenario analysis**	**Government perspective**	**Societal perspective**	**Government perspective**	**Societal perspective**
Low disease incidence	119	101	680	655
Low efficacy	193	177	1,079	1,058
Low treatment cost	92	88	533	529
Low vaccine coverage	89	73	530	510
Base case scenario	89	73	530	510
High vaccine coverage	89	73	530	510
High treatment cost	89	73	530	510
High efficacy	89	73	530	510
High disease incidence rate	89	73	530	510
**Probabilistic sensitivity analysis**				
Median ICER	91	73	550	524
Lower 95%	68	45	433	405
Upper 95%	144	128	747	716

The results also show that the most influential parameters are vaccine efficacy, disease incidence and treatment costs, in descending order. Low vaccine efficacy resulted in ICERs of USD 193 and USD 177 respectively from the perspectives of government and society in the period of 2012–2025. It also resulted in ICERs of USD 1,079 and USD 1,058 respectively from the perspectives of government and society in 2026–2031.

It can also be deduced from the results that low vaccine efficacy led to an increase in the ICER whereas high vaccine efficacy led to a decrease in the ICER. This implies that for the vaccination programme to be more cost-effective, the vaccine efficacy needs to be high. The results from the scenario analysis also show that the cost per DALY averted even from the most unfavorable scenario to the vaccine is highly cost-effective when compared to the 2022 0.5 of GDP per capita of Ghana.

### 3.6 Probabilistic sensitivity analysis

Table 6 shows the results of the probabilistic sensitivity analysis (PSA) that was undertaken with 1,000 iterations for each of the scenarios. The parameters used to account for uncertainties in the model include vaccination coverage, vaccine efficacy, disease burden and treatment costs. For the 2012–2025 period, the median ICER is USD 91 with 95% Credible Range (CR) of USD 68–USD 144 per DALY averted. This represents 2.8%−5.9% of GDP per capita from the government perspective. The median ICER from the perspective of society is USD 73 with 95% CR of USD 45–USD 128 per DALY averted representing 1.8%−5.2% of GDP per capita. In the case of the 2026–2031 period, the median ICERs is USD 550 with 95% CR of USD 433–USD 747 per DALY averted representing 17.7%−30.5% of GDP per capita from government perspective. However, the median ICER is USD 524 with 95% CR of USD 405–USD 716 per DALY averted which represents 16.6%−29.3% of GDP per capita from the societal perspective.

**Table 6:** Two-way sensitivity analysis.

**Price per dose**	**Low vaccine efficacy**	**High vaccine efficacy**	**Low treatment cost**	**High treatment cost**	**Low vaccine coverage**	**High vaccine coverage**
2026–2031	Government (Societal)	Government (Societal)	Government (Societal)	Government (Societal)	Government (Societal)	Government (Societal)
USD 2.75	241 (221)	111 (91)	115 (110)	517 (431)	111 (91)	111 (91)
USD 14.5	1,026 (1,006)	405 (385)	533 (529)	490 (404)	504 (483)	531 (511)

The results obtained from the PSA support the evidence from the base case and scenario analyses that the PCV13 vaccination programme in Ghana is highly cost-effective from the perspectives of government and society even 6 years after the withdrawal of co-financing support by Gavi.

**Figure 2:**
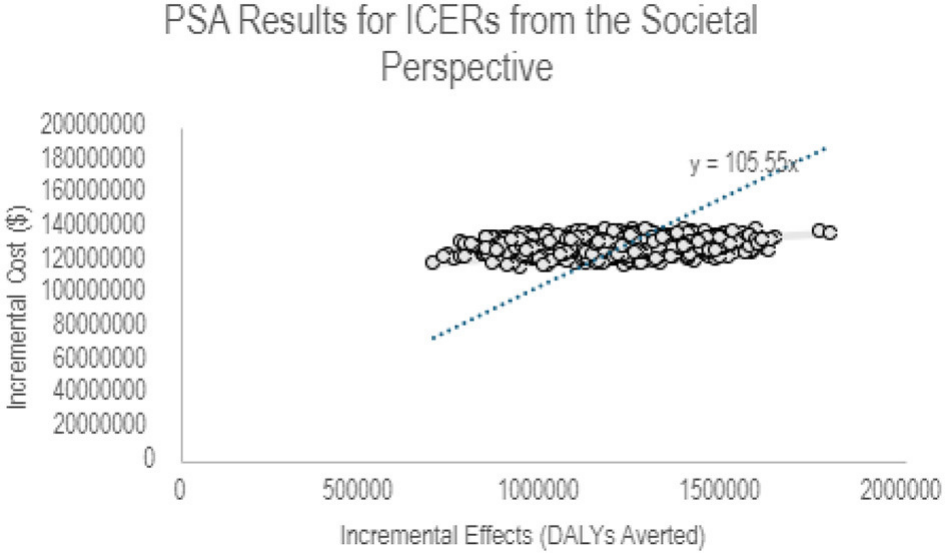
PSA results for ICERs from the societal perspective.

Figure 2 illustrates a scatter plot of the relationship between the incremental costs and the health benefits of PCV vaccination in Ghana from a societal perspective. This was obtained from 1,000 runs of the PSA in the UNIVAC model. All points lie in the NE quadrant indicating higher incremental costs of PCV vaccination for more DALYs averted at a willingness to pay threshold of USD 105.55.

### 3.7 Two-way sensitivity analysis

When the price per dose increases to USD 14.5, it results in higher ICERs than a price per dose of USD 2.75 in the period of 2026–2031. Hence, the ICER rises to USD 1,026 and USD 1,006 per DALY averted from the government and societal perspectives respectively, when the price per dose increases to USD 14.5 with a low efficacy as shown in Table 5. However, in evaluating a low vaccine price per dose against vaccine efficacy, treatment cost or vaccination coverage will result in USD 111 and USD 91 per DALYs averted respectively from the government and societal perspectives in three of the scenarios. The only exceptions are low vaccine efficacy and high treatment costs. In the case of higher treatment cost, it results in the highest ICER of USD 517 and USD 431 per DALY averted from the perspectives of government and society respectively. Since there is certainty in the price per dose during the period of 2012–2025, there was no need to conduct a two-way sensitivity analysis for that timeframe.

Table 6 shows results of assessing PCV13 price per dose against vaccine efficacy, vaccination coverage and treatment costs from a two-way sensitivity analysis. PCV13 vaccination is highly cost-effective in all the scenarios evaluated.

### 3.8 Budget impact analysis

Table 7 shows a budget impact (undiscounted) of the PCV13 vaccination program from 2026 to 2031, the first 6 years of the withdrawal of co-financing support from Gavi. The net budget impact is over USD 280 million for the period under consideration from the perspective of government. This is greater than the net cost of vaccination of over USD 275 million. It also gives a clearer picture of how much the Government of Ghana needs to budget in terms of non-wage resources to finance the PCV13 vaccination program to achieve universal health coverage by 2030.

**Table 7:** Budget impact of PCV vaccination.

**Budget impact analysis**			
**Year**	**Vaccination program cost (USD)**	**Health system cost (USD)**	**Difference**
2026	48,475,584	2,513,196	45,962,388
2027	48,835,019	2,531,831	46,303,188
2028	49,149,129	2,548,116	46,601,013
2029	49,473,328	2,564,924	46,908,404
2030	49,844,053	2,584,144	47,259,909
2031	50,278,953	2,606,691	47,672,262
Net budget impact (USD)			280,707,164

## 4 Discussions

It is estimated that PCV13 vaccination can avert about 3.7 million and about 1.7 million cases of diseases associated with Streptococcus pneumonia over the periods 2012–2025 and 2026–2031 respectively. Cumulatively, the PCV13 vaccination programme is estimated to avert about 48% of the disease burden of Streptococcus pneumonia in Ghana from 2012 to 2031. These findings align with the results of a study undertaken in Accra and Tamale which revealed PCV13 to have 48%−51% coverage of pneumococcal isolates in Ghana (26). Similar findings were also obtained from a study in the Islamic Republic of Iran where PCV13 vaccination would avert over 4.5 million cases and 38% of deaths from 2014 to 2023 (27). The findings of our study also corroborate many studies in LMICs. In assessing the health effects of PCV in children < 5 years for 30 birth cohorts (2015–2045), results from 180 countries show that 12% of global investments are required to introduce PCV in the whole of Africa. This will save 69% of lives and avert 63% of DALYs on a global scale (7).

The total costs of introducing PCV13 programme in Ghana are approximately USD 130 million and USD 275 million for the periods of 2012–2025 and 2026–2031 respectively. The incremental health system costs of PCV13 vaccination are approximately USD 2 million and USD 2.6 million for the periods of 2012–2025 and 2026–2031 respectively. Hence, Ghana is expected to incur more than twice the cost of PCV13 vaccines for the next 6 years after Gavi exits as compared to the 14 years of co-financing support from Gavi. This is mainly attributable to a sharp increase in the price per dose of PCV13 from USD 2.75 to USD 14.50. The Government of Ghana would spend an additional USD 145 million representing 53% increase to purchase PCV13 in the years following Gavi transition compared to the 14 years of partnership with Gavi. This finding is comparable to the study of Vodicka et al. (28) that Ghana's share of Gavi co-financing of price per dose will increase from 32% to 100% as the country moves toward Gavi full self-financing phase.

Furthermore, the societal cost of Streptococcus pneumonia during 2012–2025 is USD 79.3 million, out of which USD 39.7 million (50%) would be averted by vaccination. The cost to the government within the same period is USD 38.4 million, out of which USD 18.5 million (48%) would be averted by vaccination. During the 2026–2031 period, the economic burden to society is USD 41 million, out of which USD 19.6 million (49%) would be averted by vaccination. In the same period, government will avert USD 9.5 million (48%) out of a cost of USD 19.9 million through vaccination. It is noteworthy that, the PCV13 vaccination programme in Ghana is not necessarily a cost-saving intervention. Thus, the vaccine programme costs are greater than the government cost savings attributable to averted disease. This finding is consistent with the assertion of a previous study in Kenya which argues that continuing the PCV programme after transitioning from the support of Gavi is highly cost-effective although not cost-saving (29). In contrast however, a study in The Gambia reports that the PCV programme is likely to be cost-saving between 2011 and 2030, with projected reductions in disease burden and savings of USD 4 million for medical care (8). The findings also indicate that the PCV programme will remain cost-saving until The Gambia pays approximately USD 0.66 per dose (8).

### 4.3 Cost-effectiveness of PCV vaccination in Ghana

At a discount rate of 3%, results from the base case analysis find ICERs to be USD 118 and USD 97 per DALY averted respectively from government and societal perspectives in 2012–2025; and USD 530 and USD 510 per DALY averted respectively from government and societal perspectives in 2025–2031.

Each of the ICERs from the two time frames and perspectives are < 50% of the 2022 GDP per capita of Ghana (USD 1,222.5), hence could be considered cost-effective. However, the WHO thresholds have been a subject of intense academic debate in recent times (30). Meanwhile, Ghana currently does not have an established threshold to determine the cost-effectiveness of an intervention. Nonetheless, contemporary econometric modeling based on opportunity costs and income elasticity recommends probable cost-effectiveness thresholds of 4%−40% GDP per capita for Ghana (25). Moreover, recent recommendations on ICER thresholds have been to consider 0.5 × GDP per capita (31). A similar study on the cost-effectiveness of Rotavirus vaccination in Ghana used the 0.5 × GDP per capita as the ICER threshold (32). Based on these two models on ICER threshold, the study shows that PCV program in Ghana gives good value for money from the perspectives of both government and society. This is plausible under a scenario when Ghana fully transitions from the co-financing support of Gavi from 2026. Our findings are also similar to other national level studies on PCV13 vaccination where the UNIVAC model was used to conduct cost-effectiveness analyses in Nigeria, India, and The Gambia (8, 33, 34).

Comparing with the cost-effectiveness of other vaccination programmes where UNIVAC was used for modeling, PCV13 is more cost-effective (USD 97–USD 118 per DALY averted) in the period that Ghana is in partnership with Gavi than Rotavirus vaccination (USD 238–USD 360 per DALY averted) and Human Papillomavirus (HPV) vaccination (USD 152–USD 488 per DALY averted) in Ghana (10, 28, 32). However, PCV13 is less cost-effective (USD 510–USD 530 per DALY averted) if Ghana assumes full responsibility of purchasing the vaccines from 2026. These differences could be attributed to variation in model inputs such as vaccine price per dose, vaccine efficacy, disease burden, disability weights, and cost of treatment, among others. The huge differences could also be as a result of the modeling approach used in the various studies. Whereas Nonvignon et al. (10) estimated costs under a scenario of the price per dose Ghana pays for the Rotavirus vaccine whilst benefiting from Gavi co-financing, and a second scenario where Ghana pays fully for the vaccines for 20 years in either cases; this study rather modeled based on the price per dose borne by Ghana during the period (2012–2025) of partnership with Gavi and separately for the period that Ghana might pay fully for the vaccine after the exit of Gavi support (2026–2030).

In a context where many LMICs including Ghana are on the verge of paying fully for vaccines after the exit of Gavi and are facing economic challenges, it is essential to assist policy-makers in the efficient allocation of resources through cost-effectiveness analysis. Hence, policy-makers and development partners could make a strong investment case using the results from this study to sustain the PCV13 vaccination programme as it is currently the most potent public health tool to combat Streptococcus pneumonia in Ghana. It would also serve as a guide for GAVI, UNICEF and other development partners to prioritize their country programmes on childhood vaccination.

The UNIVAC model used in this study has some limitations. First of all, as a static model, it assumes that there will be no change in the population that is susceptible to infection of Streptococcus pneumonia. Thus, it does not consider complicated health transition states of the disease. Also, the model does not take indirect effects or herd protection of the vaccine on the unvaccinated population into consideration and hence, the cost-effectiveness might be underestimated (28, 35). Furthermore, the number of cases were based on published literature and international estimates. This also has the potential to underestimate the disease burden of Streptococcus pneumonia due to low case detection and instances where some children might have died before their specimens were taken to the laboratory for confirmation (34). However, the model has several advantages that allowed for its use in this study. Some of these include its transparency and ease of interpretation to policy makers, use of minimal set of data inputs, ability to make standardized comparison between different vaccine policies and flexibility as it can easily be adapted to assess interventions in a judicious manner (10, 14).

## 5 Conclusion

In spite of variations in the results of the scenarios in terms of costs and ICERs, the findings consistently indicate that PCV13 vaccination is a high impact and highly cost-effective intervention in Ghana. However, it is not necessarily cost-saving. Hence, PCV13 vaccination gives value for money under the cost-effectiveness threshold of 0.5 × GDP per capita. In a context where many LMICs including Ghana are on the verge of paying fully for vaccines after the exit of Gavi and are facing economic challenges, it is essential to assist policy-makers in the efficient allocation of resources through cost-effectiveness analysis. Hence, policy-makers and development partners could make a strong investment case using the results from this study to sustain the PCV13 vaccination programme as it is currently the most potent public health tool to combat Streptococcus pneumonia in Ghana.

## Data availability statement

The original contributions presented in the study are included in the article/supplementary material, further inquiries can be directed to the corresponding author.

## Author contributions

A-MI: Conceptualization, Data curation, Formal analysis, Methodology, Writing – original draft, Writing – review & editing, Project administration. RO: Conceptualization, Data curation, Formal analysis, Methodology, Writing – original draft, Writing – review & editing, Supervision, Validation. JN: Conceptualization, Supervision, Validation, Writing – review & editing.
